# Macro Histone Variants: Emerging Rheostats of Gastrointestinal Cancers

**DOI:** 10.3390/cancers11050676

**Published:** 2019-05-15

**Authors:** Sebastiano Giallongo, Oriana Lo Re, Manlio Vinciguerra

**Affiliations:** 1 International Clinical Research Center, St. Anne’s University Hospital, 65691 Brno, Czech Republic; sebastiano.giallongo@fnusa.cz (S.G.); oriana.lore@fnusa.cz (O.L.R.); 2 Department of Biology, Faculty of Medicine, Masaryk University, 62500 Brno, Czech Republic; 3 Institute for Liver and Digestive Health, Division of Medicine, University College London (UCL), London NW32PF, UK

**Keywords:** gastrointestinal cancer, histone variants, epigenetics, macroH2A

## Abstract

Gastrointestinal cancers (GC) are malignancies involving the gastrointestinal (GI) tract and accessory organs of the digestive system, including the pancreas, liver, and gall bladder. GC is one of the most common cancers and contributes to more cancer-related deaths than cancers of any other system in the human body. Causative factors of GC have been consistently attributed to infections, smoking, an unhealthy diet, obesity, diabetes, and genetic factors. More recently, aberrant epigenetic regulation of gene expression has emerged as a new, fundamental pathway in GC pathogenesis. In this review, we summarize the role of the macroH2A histone family in GI cell function and malignant transformation, and highlight how this histone family may open up novel biomarkers for cancer detection, prediction, and response to treatment.

## 1. Introduction

### 1.1. Gastrointestinal Cancer Epidemiology

Gastrointestinal cancer (GC) refers to malignant conditions of the gastrointestinal (GI) tract and accessory organs of the digestive system, including the esophagus, stomach, biliary system, pancreas, small intestine, large intestine, rectum, and anus. GC symptoms vary depending on the organ(s) affected, and can include gastric obstruction, abnormal bleeding, or other associated issues. GC is typically diagnosed by endoscopy, accompanied by biopsy to determine the nature of the suspicious tissue. GC accounts for ~20% of new cancer cases and ~15% of cancer-associated deaths worldwide [1]. Overall, the GI tract and accessory organs host more cancers than any other system in the body, and these cancers make the highest contribution to cancer-associated mortality. Specifically, esophageal cancer is the sixth most common cancer world-wide; stomach (or gastric) cancer is the fourth most common; pancreatic cancer is rare but is the fifth most common cause of cancer-associated deaths; and liver and gallbladder cancers are relatively infrequent but carry a very poor survival rate [2]. In the lower GI tract, colorectal cancer is the second most common cancer, constituting ~10% of all cancer cases, while anal cancer constitutes only 1% of all cancer cases and has a high survival rate [2]. Interestingly, the prevalence of different GCs shows marked geographic variation [3]. Over the past 20 years, the burden of cancers attributable to being overweight (body mass index (BMI) between 25 and 30), obesity (BMI > 30), and diabetes mellitus has markedly increased, particularly in low-income and middle-income countries [4].

Strong epidemiological evidence linking adiposity with GC outcomes has derived from a plethora of studies conducted on North American, European and Asian populations [5,6]. Now, several complex mechanistic pathways have been proposed to underlie the obesity–GC link. These pathways involve systemic inflammation, metabolic reprogramming, oxidative stress, DNA repair, cell death, angiogenesis, the gut microbiome, and immune function [7]. High-throughput analytical omic approaches, such as genomics, epigenomics, transcriptomics, proteomics, and metabolomics, are increasingly being combined with epidemiological studies to identify new therapeutic targets for GC [6]. Analyzing biological samples using such omic technologies prior to diagnosis has the potential to provide new insights into how obesity is associated with GC development. Moreover, earlier detection and improved prognosis have the potential to reduce the overall mortality burden from GI cancers. [8].

### 1.2. Gastrointestinal Cancer and Epigenetics

Epigenetic changes, including aberrant DNA methylation, are common events in GC initiation and progression [9]. Consequently, DNA methylation levels are emerging as promising biomarkers for early GC detection, prognosis, and development, and for predicting response to therapy [9]. Now, an active area of investigation is whether such epigenetic changes may be identified non-invasively using body fluid samples [10]. Other epigenetic alterations include histone modifications, histone variants, loss of genome imprinting, chromatin remodeling and the activity of non-coding RNAs, all of which are broadly associated with carcinogenesis in humans [11]. Histone variants—notably canonical histone H2A and H3—are increasingly appreciated to endow chromatin with unique properties. Such variants have important roles in regulating stem cell lineage commitment and reprogramming somatic cells to a state of pluripotency [12]. Changes in histone variant deposition onto chromatin affect tumorigenesis by modulating chromatin plasticity, genomic instability and cellular senescence, and triggering cancer-promoting gene expression pathways [12]. In this review, we summarize the current knowledge on the role of macroH2A histone family members as biomarkers and drivers of cancer pathogenesis in the GI tract.

## 2. The MacroH2A Histone Family Variants

Chromatin is a complex composed of histones, DNA, and proteins that is localized in the cell nucleus. The basic repeating unit of chromatin, known as the nucleosome, consists of 146 base pairs of DNA wrapped around an octameric core of “canonical” histones (H2A, H2B, H3, H4); this unit is crucial to all DNA-based physical–chemical phenomena [13]. Histone H1 binds to linker DNA sequences that are ~20–80 nucleotides in length, to connect two adjacent nucleosomes. Nucleosomes allow for genome compaction and protection in the nucleus, and their composition and post-translational modification is responsible for regulating gene expression [14]. In addition to the canonical histones that comprise the bulk of histones found in the cell, histone variants with specific properties have emerged over the course of evolution. H2A (H2A.X, H2A.Z.1, H2A.J, H2A.Z.2.1, H2A.Z.2.2, H2A.Bbd, macroH2A1.1, macroH2A1.2. and macroH2A2) and H3 (H3.3, CENP-A, H3.1, H3.2, H3T, H3.5, H3.X. and H3.Y) variants have been identified in human somatic cells, and H2B variants (H2BFWT and TSH2B) have been identified in germ cells [12]. To date, no H4 variants have been discovered in humans or other higher eukaryotes [12]. The diversity and evolution of histone variants and their biological role has been covered in other recent reviews [15,16,17,18,19].

Histone variants differ in terms of their genetic sequence, the timing and modality of processing from RNA to mature protein, and chromatin deposition during the cell cycle [12]. While the coding genes for canonical histones are organized into clusters, histone variants are typically only encoded by one or two genes. The unique temporal pattern of expression of each histone variant influences its specific cellular functions [12]. A degree of diversity among histone variants is also conferred by the presence of introns that can be spliced during RNA processing, providing the opportunity to generate alternative splice isoforms and increase transcriptional efficiency.

**Figure 1:**
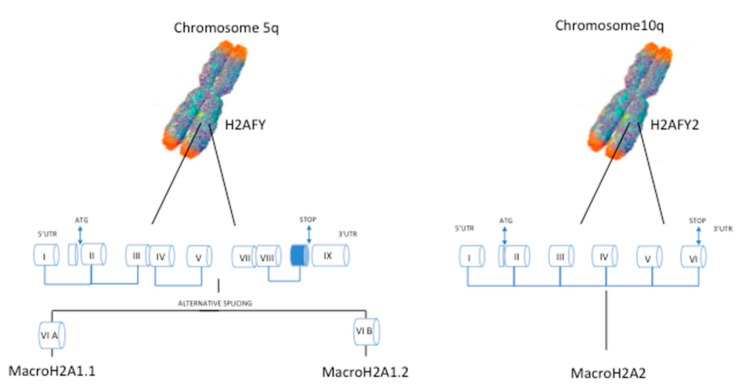
Representation of the chromosomal location of macroH2A1 and macroH2A2 genes in humans, and the organization of exons in the gene bodies. MacroH2A1 mRNA gives rise to two alternatively spliced isoforms, macroH2A1.1 and macroH2A1.2.

One example of histone variant proteins generated by alternative splicing is macroH2A1, which was described in 1992 by Pehrson et al. [20]. MacroH2A1 contains a domain that shows 66% homology with histone H2A and is conserved across various functionally unrelated proteins throughout the animal kingdom, vertebrates and some invertebrates [21]. This variant stands out because of its unique structure, whereby a C-terminal linker connects the histone fold domain to a macrodomain [22]. This macro domain protrudes from the compact nucleosome structure and likely affects the function and organization of the surrounding chromatin. Once in the nucleosome, macroH2A1 may contribute to different cellular processes, including cell cycle regulation, embryonic and adult stem cell differentiation, and DNA repair and transcription in somatic and cancer cells [23]. MacroH2A1 exists as two alternatively exon-spliced isoforms, macroH2A1.1 and macroH2A1.2 [24,25], while macroH2A2 is encoded by an independent gene (Figure 1).

Historically, macroH2A1 has been implicated in female X chromosome inactivation and transcriptional repression [26,27,28], as it is enriched and distributed uniformly along the condensed inactive X chromosome. Indeed, the generation of embryonic stem cells over-expressing a macroH2A1.2-GFP (green fluorescent protein) transgene has permitted the non-invasive visualization of X chromosome inactivation in mouse embryos pre-implantation [29,30]. Current evidence also supports a tumor-suppressive role for macroH2A1.1, while the role of macroH2A1.2 is dependent on the specific cancer context [12,23]. Ectopic macroH2A1 over-expression reduces the metastatic potential of melanoma and hepatocellular carcinoma (HCC) [31,32,33], whereas macroH2A1 depletion increases the aggressiveness of HCC, teratoma, and breast cancer cells [32,34,35]. This finding could be because loss of macroH2A1 enhances stem-like properties in cancer cells, as observed in the bladder [32,36]. In general, macroH2A1.1 levels inversely correlate with proliferation. In fact, this variant is downregulated in many cancer types, and this down-regulation is associated with a poor prognosis [37,38]. Alternative splicing of macroH2A1 isoforms, however, does not occur in all tumor types; it seems irrelevant in HCC, for instance, where down-regulation of both macroH2A1.1 and macroH2A1.2 occurs at the mRNA and protein levels [31,32,39,40].

## 3. MacroH2A and GC of the Upper Digestive Tract

The upper digestive tract comprises the oral cavity, the esophagus (fibro-muscular tube where food passes via peristaltic movements), and the stomach. Several histological tumor types occur in this anatomical area. The two most common esophageal cancer histological types are squamous carcinoma and adenocarcinoma, which are both associated with a high mortality rate [41]. Stomach cancer is more aggressive than esophageal cancer; it is the fourth most common cause of cancer-related deaths worldwide and it usually originates from GI stromal tissue involving the proximal stomach and the gastro-esophageal junction [42].

**Figure 2:**
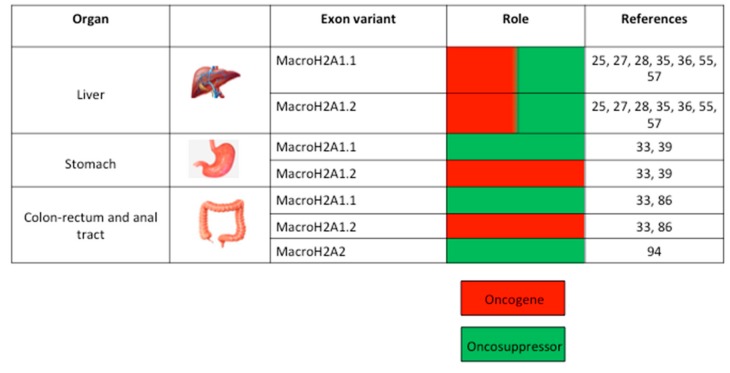
Representation of the role of macroH2A histone variant family members (macroH2A1.1, macroH2A1.2, macroH2A2) in tumorigenesis in different parts of the gastrointestinal (GI) tract.

As mentioned, macroH2A1 mRNA is processed through alternative splicing to produce macroH2A1.1 and macroH2A1.2 isoforms. This process is modulated by the expression of splicing factors, including various members of the Quaking (or QKI) protein family [37]. The QKI5 RNA binding factor was originally identified by Novikov et al., who, through an informatics approach, showed that it was one of the proteins involved in macroH2A1 alternative splicing [37]. Specifically, they identified a direct correlation between QKI5 expression and macroH2A1.1 levels. QKI protein expression in patients with GC of the upper digestive tract was later studied by Li et al. [43]. Here, the researchers found an overall down-regulation of QKI family members in cancer cells, including QKI5 (the dominant isoform in GC cells), which mostly localizes to the nucleus. Li et al. found increased macroH2A1.1 expression in HGC-27 GC cells, in which QKI5 was ectopically over-expressed and downregulated in GC tissue where QKI5 was depleted [43]. Conversely, macroH2A1.2 expression showed a negative correlation with QKI5 levels: it was downregulated in cells over-expressing QKI15 and up-regulated in GC clinical tissues [43]. Functional assays identified a tumor suppressive role for macroH2A1.1 in GC: Cyclin dependent kinase 8 (CDK8) expression analysis and wound healing assays showed that HGC-27 cells over-expressing macroH2A1.1 were less proliferative and invasive than control cells [43]. Moreover, QKI5 or macroH2A1.1 over-expression in GC cells led to down-regulated expression of the oncoprotein cyclin L1, which has been directly associated with poor survival in patients with GC [43]. Taken together, these data support that macroH2A1.1, but not macroH2A1.2, functions as a tumor suppressor protein in GC via a QKI5–macroH2A1.1–cyclin L1 (CCNL1) axis (Figure 2). 

## 4. MacroH2A and GC of the Hepatobiliary Tree

The hepatobiliary tree includes the liver, gallbladder, and bile ducts [44]. These organs work together to produce and secrete bile, the components of which are synthesized by hepatocytes and extracted directly from blood [45]. Gallbladder carcinoma, cholangiocarcinoma, and HCC are the main malignancies to occur in the hepatobiliary tree [46]. Of these, HCC is the most prevalent and is the second most frequent cause of cancer-associated death worldwide [47]. A prominent risk factor for HCC development is hepatitis virus (HV) B or C infection, which contributes to ~85% of all HCC cases [48]. Cirrhosis is another important risk factor in HCC pathogenesis, with ~33% affected patients developing HCC in their lifetime; cirrhosis can occur due to chronic HV infection, alcohol abuse, or lifestyle-dependent metabolic dysfunction, such as non-alcoholic fatty liver disease (NAFLD) [49].

### 4.1. MacroH2A1 and NAFLD

NAFLD is an accumulation of intra-hepatic triglycerides that is often considered the hepatic manifestation of insulin resistance [50]. NAFLD is widespread in Western countries, with up to one third of the population being affected; in the USA, it is the most common form of chronic liver disease, affecting an estimated 80–100 million people [50]. NAFLD is a spectrum of disturbances that encompasses various degrees of liver damage, ranging from simple steatosis to non-alcoholic steatohepatitis (NASH). NASH is characterized by hepatocellular injury/inflammation with or without fibrosis. In cases where the inflammation becomes persistent, there is an increased probability of developing fibrosis, characterized by scar tissue around the liver and blood vessels. In the context of obesity and NAFLD, epigenetic events have a role in chromatin remodeling and plasticity in hepatocytes [50], as discussed below.

A link between histone macroH2A1 and NAFLD was demonstrated in 2011 by Chalgonkar et al., who developed a thiol-affinity-based method to isolate macroH2A1 nucleosomes from female mouse liver chromatin [51]. Once isolated, the researchers purified and sequenced the DNA associated with the nucleosomes and mapped the genes interacting with macroH2A1. They found that genes implicated in lipid metabolism, including *Lpl*, *Vldlr*, *Cd36*, *Scd2*, *Acot1*, and *Acot2*, were associated with macroH2A1, indicating that macroH2A1 is somehow involved in this pathway. At the subcellular/nuclear level, Yuhua et al. attempted to understand the role of macroH2A1 in hepatocyte chromatin architecture by investigating the association of this protein with lamina-associated domains (LAD). The nuclear lamina is a dense fibrillar network that regulates the nuclear structure and is involved in various biological processes [52]. During interphase, ~40% of the mammalian genome is organized into LAD. Using mouse liver cells as an in vitro model, Yuhua et al. showed that macroH2A1 associated with the LAD boundaries, which coincided with H3K27me3 expression, a marker of inactive genes. Silencing macroH2A1 led to global chromatin decondensation, indicating that this protein is required for stabilizing the chromatin architecture in mouse liver cells [53].

At the level of hepatic pathophysiology, we have analyzed the role of macroH2A1 in NAFLD development. To study the expression of both macroH2A1 isoforms and their involvement in NAFLD, we considered two different mouse models of NAFLD/NASH/HCC [40]: a high fat diet (HFD)/diethylnistrosamine (DEN) mouse, and a phosphatase and tensin homolog (PTEN) liver-specific knock-out (KO) mouse [40]. We found that in HFD/DEN-treated mice, macroH2A1.1 and macroH2A1.2 protein expression levels increased during HCC development compared to control mice. However, only macroH2A1.2 expression increased in the mouse liver developing NAFLD. In the PTEN KO mice, which paradoxically display hepatic insulin hypersensitivity and increased systemic glucose tolerance [54], we again observed a significant increase in macroH2A1.1 and macroH2A1.2 protein expression in the HCC setting. As before, macroH2A1.2 but not macroH2A1.1 protein expression was enhanced specifically in the livers of 16 week old PTEN KO mice developing NAFLD, compared to age-matched control PTEN flox/flox mice. This finding indicates that macroH2A1.2 might be an epigenetic marker of NAFLD [40].

Several whole body macroH2A1 (whole gene) KO mice have been developed to demonstrate a role for this histone protein in lipid metabolism and obesity [55]. We recently reported that C57BL/6 macroH2A1 KO mice fed a HFD exhibited ~10% reduced weight gain compared to wild-type (WT) mice, due to a decrease in body-fat mass [56]. Moreover, WT but not KO mice showed periportal inflammation with an accumulation of lymphocytes upon a HFD, highlighting a protective effect of a systemic lack of macroH2A1 [56]. These KO mice also showed reduced heat production and increased glucose tolerance with enhanced insulin sensitivity in the skeletal muscle but not liver. These findings diverge somewhat from those of earlier studies that rather showed that loss of macroH2A1 worsens lipid metabolism, reviewed in Reference [55].

Chalgonkar et al. originally described the constitutive macroH2A1 KO mouse model, generated on a C57BL/6 background [51,57]. A mild effect due to the lack of macroH2A1 was reported in several organ systems; in particular, KO mice displayed an enlarged spleen with increased lymphocyte infiltration, and mild inflammation of various tissues. Gene expression profiling in newborn and adult female livers from these KO mice showed an increase in *Lpl, Scd2, Thrsp*, and *CD36* levels, which favors NAFLD development [51,57]. These mice also developed glucose intolerance and insulin resistance [51,57]. Sexual dimorphism was also observed in these KO mice; female mice showed a small increase in blood glucose concentrations compared with male mice, suggesting that this mechanism may be due to a differential response to increased fatty acid delivery to the liver between sexes. Finally, changes in lipogenic gene expression were found to correlate with genomic occupancy by macroH2A1 [51,57].

Boulard et al. developed an alternative macroH2A1 KO mouse model by intercrossing the 129Ola x C57Bl/6 genetic backgrounds: here, they observed an up-regulation of *Tbg* (X-linked thyroxine-binding globulin) in steatotic female livers [58]. This protein is a carrier of Thyroid T4 and is involved in various metabolic pathways. The researchers noted that loss of macroH2A1 correlated with *Tbg* up-regulation, leading to altered lipid metabolism and lipid accumulation in female mice during hepatic steatosis development [58]. While these studies support that macroH2A1 is involved in systemic and hepatic lipid metabolism [55], they did not provide insights into the differential roles of the macroH2A1.1 and macroH2A1.2 isoforms.

Further evidence for a macroH2A1-isoform specific role in hepatic lipid accumulation has come from in vitro models using human and murine hepatic cell lines. For example, we have shown that ectopic macroH2A1.1, but not macroH2A1.2, over-expression in human and mouse hepatocytes increases glycogen synthesis and glucose uptake [59]. This effect confers protection against lipid accumulation, and triggers decreased expression of genes involved in fatty acid synthesis/transport and the metabolism and transport of cholesterol [59]. We observed a completely opposite pattern upon ectopic macroH2A1.2 over-expression in the same cell lines, even upon free fatty acid (FFA) treatment [59]. Together, these data imply that the adenosine diphosphate ribose (ADP)-ribose binding module specific to macroH2A1.1 is required for its anti-lipidogenic effects.

### 4.2. MacroH2A1 and Adipogenesis

Wan et al. investigated the role of the macroH2A1.1 isoform in adipogenesis using 3T3-L1 cells [60]. They showed that macroH2A1.1 levels increased during adipogenesis, while macroH2A1.1 knockdown inhibited adipogenesis. The same evidence has not been found for macroH2A1.2, thus implying the specificity of macroH2A1.1 in this process. These in vitro data are supported by in vivo studies performed in C57Bl/6 mice: mice fed a HFD, but not a control diet, showed drastically increased macroH2A1.1 levels, but macroH2A1.2 levels were unchanged [60].

Podrini et al. investigated the role of both macroH2A1.1 and macroH2A1.2 in FFA accumulation in HepG2 and immortalized human hepatocyte cells [61]. The researchers confirmed that over-expression of macroH2A1.1, but not macroH2A1.2, led to a decreased level of triglycerides and lipid peroxidation in hepatic cell lines. Moreover, upon FFA administration, macroH2A1.1 over-expression decreased the transcription of genes involved in lipogenesis. Conversely, knockdown of the whole H2AFY transcript by siRNA resulted in the down-regulation of genes involved in FFA intake, including *FATP*2 and *FATP4*, and lipogenic genes, including *SCD, FASN*, and *VLDLr* [61]. Finally, the researchers studied metabolic disturbances in two mouse models carrying KO first conditional-ready alleles for *Atp5a1* or *Fam73b*—two proteins implicated in metabolic defects. Both KO lines exhibited a decrease in body fat compared to WT littermate controls when fed a HFD. This phenotype was concomitant with increased macroH2A1.1 expression in the liver, proving its protective role against fat accumulation [61].

As >90% of obese subjects display NAFLD, a few studies have analyzed the role of macroH2A1 isoforms in adipose tissue in vivo. Such studies have shown that macroH2A1.1, but not macroH2A1.2, expression is increased in visceral adipose tissue biopsies from morbidly obese subjects (or mice) compared to tissues from normal weight subjects (or mice) [29,60]. Mechanistic insights underlying this finding have come from studies using a mouse model expressing a whole-body ectopic macroH2A1.2 GFP-coupled transgene [29]. In the context of a HFD, these mice exhibited decreased visceral fat accumulation, increased glucose tolerance and insulin sensitivity, and decreased hepatic and pancreatic fat accumulation and inflammation compared to WT littermates [29]. This work confirmed a protective role of macroH2A1.2 in terms of metabolic health and inhibition of adipogenesis.

The same researchers also focused on the role of the macroH2A1 isoforms during adipogenesis in vitro [29]. During the first six days of adipogenesis in 3T3-L1 cells, they observed a gradual increase in the expression of both macroH2A1.1 and macroH2A1.2 proteins. However, macroH2A1.2 levels decreased eight days after the induction of adipogenesis [29]. Furthermore, macroH2A1.1, but not macroH2A1.2, knockdown inhibited adipogenesis.

There are clear discrepancies between the in vitro and in vivo data regarding the role of macroH2A1 isoforms in intracellular lipid accumulation. This contradiction could be because this histone variant might have distinct tissue-specific functions in the liver and adipose tissue, and the lipid content in the liver is dependent on obesity/BMI, and hence the amount of lipids in excess that are mobilized from the adipose tissue. In fact, lipid accumulation in the liver can come from three sources: (i) diet, (ii) de novo synthesis, and (iii) adipose tissue [62]. The flux of FFA through the human circulation amounts to ~100 g/day, with 20% being extracted by the liver. It has been estimated that the daily input of triglycerides from the diet (~20 g/day) and FFA from adipose tissue (~20 g/day) approximates the entire lipid accumulation of the liver [62]. Moreover, the murine genetic background must be taken into account when studying the effects of macroH2A KO. For example, when macroH2A is deleted on a CB57Bl/6 background, the transgenic mice show impaired reproductive capacity and increased rate of peri-natal death that is not evident on a 129/S6 background. Conversely, 129/S6, but not CB57Bl/6 KO, mice show signs of bilaterally increased palpebral fissure, eyelid inflammation, and a darker back compared to WT littermate controls [63]. Finally, the nutritional status of these mice also has a strong impact on the effect of macroH2A1 on gene expression. During fasting, genes that are implicated in lipid metabolism, such as *Fabp5* and *Rgs16,* are altered in the livers of adult macroH2A KO mice, while their expression is almost comparable with WT littermates under normal feeding conditions [63]. The generation and study of adipose tissue-specific or liver-specific macroH2A1.1/macroH2A1.2 KO or transgenic mice will help to solve these discrepancies regarding their specific in vivo impact on nutrient metabolism.

### 4.3. MacroH2A1, Methylation Status, and HCC

As discussed, NAFLD together with other metabolic symptoms and cirrhosis are the leading factors triggering aging-related liver diseases. These diseases are characterized by a prominent state of inflammation that can activate tumorigenesis and promote HCC onset [64]. Molecular analyses have identified altered epigenetic processes in HCC, namely promoter-specific hypermethylation and global DNA hypomethylation [65].

Because macroH2A1 levels in the liver change with aging, it is important to investigate its role in HCC. Our lab recently studied the interplay between macroH2A1 and the epigenetic alterations that characterize HCC onset [31]. Using immunohistochemical analyses, we showed that HCC human samples expressed higher macroH2A1.1 and macroH2A1.2 levels compared to healthy control samples. Moreover, we detected DNA hypomethylation along the whole liver disease spectrum, with a correlation between epigenetic changes occurring in HCC and macroH2A1 isoform expression [31]. An altered DNA methylome is one contributing factor that can lead to HCC tumorigenesis [66]. Treatment of HCC cells with decitabine, a chemotherapeutic that induces DNA hypomethylation, induces cell senescence and decreases tumor proliferation [31]. The researchers went on to show that HCC cells over-expressing macroH2A1.1 or macroH2A1.2 developed resistance to decitabine-induced senescence through a pathway depending on p38 mitogen-activated protein kinase/interleukin (MAPK/IL8) signaling [31]. A study by Jueliger et al., however, showed that HCC cells are sensitive to guadecitabine—a demethylating agent representing a modified version of decitabine, in which the agent is stabilized by the covalent addition of guanosine [39]. Here, macroH2A1 isoforms over-expression in HCC cells resulted in cytidine deaminase (CD) up-regulation, which degraded decitabine but not guadecitabine: this phenomenon is due to the different chemical structures rendering guadecitabine five times more resistant to enzymatic CD-dependent degradation [39].

Tumor tissues harbor a sub-population of cancer stem cells (CSCs) characterized by enhanced tumorigenic potential; these cells are responsible for tumor relapse. Because macroH2A1 has been implicated in stem cell differentiation, we recently investigated its involvement as an epigenetic factor involved in CSC insurgence and stemness [31,34]. By assaying HCC human samples by immunohistochemistry (IHC), we found a direct correlation between macroH2A1 expression and tumor differentiation. Next, to deeply understand the role of macroH2A1 in tumor differentiation, we inoculated female athymic nude mice with control (CTL), macroH2A1 knock-down (KD), or macroH2A1.1 or macroH2A1.2 over-expressing HepG2 cells. Mice inoculated with KD cells, but not CTL cells, showed larger xenograft tumors, characterized by a low differentiation status. On the other hand, tumor cells over-expressing macroH2A1.1 or macroH2A1.2 reached a smaller size compared to WT cells, supporting the idea that macroH2A1 expression is associated with HCC differentiation. Moreover, macroH2A1 KD cells showed typical CSC features: they became resistant to doxorubicin and sorafenib, two of the most used chemotherapeutics against HCC, and to hypoxia through hypoxia inducible factor (HIFα1) up-regulation. Finally, we showed that the CSC phenotype was achieved through nuclear factor kappa-light-chain-enhancer of activated B cells nuclear factor kappa-light-chain-enhancer of activated B cells (NF-κB)p65 phosphorylation at Ser536, an oncogenic driver involved in the development of several tumors [32]. An in-depth characterization of the cellular metabolism of macroH2A1 KD cells showed a significant shift toward the pentose phosphate pathway (PPP). Specifically, KD cells produced an increased amount of glucose 6-phosphate and nicotinamide adenine dinucleotide phosphate (NADPH) concomitant with increased nucleotide synthesis. Moreover, the KD cells showed an increased oxygen consumption rate (OCR)/extracellular acidification rate (ECAR), indicative of metabolic reliance on glycolytic and PPP pathways [67]. Overall, we showed that KD cells produced higher levels of acetyl-CoA, which is used as substrate in fatty acid synthesis, as mirrored by the increased number of lipid droplets found in KD cells [32,67]. We posited that the central process leading to this change is probably dependent on liver X receptors (LXRs), as we found LXR genes to be differentially regulated in KD compared to WT cells. Treating cells with SR9243, an inverse LXR antagonist, restored gene expression levels in KD cells to basal levels [67].

Taken together, these data highlight a role for macroH2A1 in HCC progression and cellular metabolism. Further studies are now required to better understand the role of macroH2A1 proteins in cancer cell stemness. 

## 5. MacroH2A and GC of the Lower Digestive Tract 

The lower GI tract comprises most of the small intestine and the entire large intestine. The small intestine is divided into the duodenum, jejunum, and ileum while the large intestine is divided into the colon and anal tract [68]. The epithelium covering the intestine is one of the most highly proliferative tissues in the human body. The high proliferation rate is achieved by the presence of two different populations of intestinal stem cells (ISC): fast-cycling crypt base columnar (CBC) and slow-cycling reserve ISCs [69]. When CBCs are lost, ISCs have a fundamental role in epithelial regeneration, triggering CBC renewal [70,71]. The identities and differentiation of the two ISC classes are mostly governed by epigenetic factors. Cedeno et al. showed that macroH2A1, but not macroH2A2, is expressed in the proximity of crypts and villi [72]. Using a double knock-out (DKO) macroH2A1 mouse, the researchers found that loss of macroH2A1 in the epithelium caused an increased number of ISCs compared to WT mice [73]. Nevertheless, these cells were less resistant to DNA damage, showing an impaired regenerative response after ionizing γ-radiation exposure, likely due to an increased rate of apoptosis.

### 5.1. MacroH2A1 and CRC Pathogenesis

The macroH2A histone family has been implicated in colorectal cancer (CRC) pathogenesis. CRC is the third most prevalent cancer worldwide, affecting women and men equally, with an incidence of 1 million of people per year and an associated mortality rate of 33% [74]. Certain genetic disorders have been associated with an increased risk of developing CRC, most of which are inherited in an autosomal dominant fashion [75]. Familial adenomatous polyposis (FAP) and Lynch syndrome (hereditary nonpolyposis CRC) are the most common genetic diseases, and together constitute ~5% of all CRC cases [76]. A higher proportion of CRC cases may be associated with an inherited syndrome that typically results in the appearance of multiple, asymptomatic adenomatous polyps [77].

MacroH2A1 was initially proposed as an oncoprotein in CRC cells. Considering the similarities with the inactive X chromosome, where the protein was originally localized [20], Barzily-Rokni et al. demonstrated macroH2A1 occupancy on the body of silenced tumor suppressor genes, including p16 [78]. Using two CRC cell lines, they also showed that siRNA-mediated macroH2A1 silencing led to decreased tumor proliferation after rescue of p16 expression, suggesting that macroH2A1 could be considered an oncoprotein [78] (Figure 2). Nevertheless, increasing evidence suggests that macroH2A1 might instead function as a potential barrier to CRC development [79]. De Barrios et al. [80] found that the gene encoding macroH2A1, *H2AFY*, is silenced by the oncoprotein Zinc Finger E-Box Binding Homeobox 1 (Zeb1). Zeb1 is a protein target of the Wnt (Wingless-related integration site) pathway, and when it is expressed in malignant cells it triggers the epithelial-to-mesenchymal transition (EMT), determining a worse clinical prognosis in most human cancers [81]. When analyzing the effect of Zeb1 ex vivo, the researchers found that its maximum effect on promoting CRC progression was achieved only upon co-expressing Dickkopf-related protein 1 (DKK1)—a Wnt protein antagonist that is transcriptionally activated by Zeb1 [80]. Co-expression of these two proteins in the SW480 CRC cell model also decreased the expression of senescence-associated genes [80]. MacroH2A1 is well known to regulate senescence onset, which is associated with cancer suppression [24,82]. Silencing both ZEB1 and DKK1 led to macroH2A1 up-regulation. The researchers showed by chromatin immunoprecipitation thatthe inhibitory effect of ZEB/DKK1 on macroH2A1 protein expression is achieved by the association of Zeb1 with two high-affinity *H2AFY* sites [80].

Immunohistochemical analyses of melanoma tissues have shown a decrease in macroH2A1 mRNA levels compared to control tissues [33]. Gene expression profiling of melanoma B16-F61 cells also found that decreased macroH2A1 levels correlate with a ≥2-fold change in the expression of cyclin-dependent kinase 8 (CDK8), a CRC oncogene [33,83]. Ohtzuka et al. found that H19, a long non-coding RNA (LncRNA) [84], also correlates with CDK8 expression levels. LncRNAs are defined as RNA fragments of at least 200 nucleotides that do not code for any protein [85]. H19 knockdown in HCT116 CRC cells decreased cell proliferation, with a significant reduction of cells in S phase, and an accumulation of cells in G1 phase. These effects on the cell cycle were associated with a strong reduction in CDK8 levels, suggesting that its expression is regulated by H19 [84]. Moreover, a strong physical association between H19 RNA and macroH2A1 has been reported and suggested to strongly repress CDK8 expression [33]. Taken together, these findings support the potential involvement of macroH2A1 in regulating CRC progression via CDK8 transcription [84].

### 5.2. MacroH2A1 Splicing in CRC

As discussed, the current data support that low levels of macroH2A1.1 correlate with a poor cancer prognosis [12,23], while the effect of macroH2A1.2 levels varies in a cancer-specific manner [12,23,24]. The process of alternative splicing is also altered in CRC. Ex vivo analyses have demonstrated that the percentage of macroH2A1.1 transcript expression is significantly reduced in primary CRC tumors compared to normal tissues; moreover, bioinformatic approaches based on microarray datasets have proven useful in identifying the factors that regulate macroH2A1 splicing [86]. Here, the most positively correlated splicing factor on the array was again the RNA binding protein Quaking (QKI), which has been implicated in tumor progression and clinical outcomes [87,88]. The interaction between QKI and macroH2A1 mRNA was also confirmed during genome-wide screening by Photoactivatable Ribonucleoside-Enhanced Crosslinking and Immunoprecipitation (PAR-CLIP) for site-specific interactions between RNA-binding factors and total RNA [89]. Low levels of QKI expression in multiple cancer types—including CRC—were associated with an increase in macroH2A1.2 levels and a concomitant increase in macroH2A1.1 levels [37]. Taken together, these findings support the idea that QKI levels affect macroH2A1 mRNA splicing [37].

Sporn et al. investigated the roles of macroH2A1 splicing isoforms in CRC [90] by IHC in CRC tissue specimens [90]. While the researchers uncovered a significant correlation between macroH2A1.1 expression and survival, they found no correlation between macroH2A1.2 and survival. The same researchers previously showed that macroH2A1.1 expression is indicative of a good prognosis in lung cancer [38]. IHC analysis on lung cancer tissues indicated a strong correlation between decreased macroH2A1.1 expression levels and increased levels of the proliferation marker Ki-67 [91]. Once again, macroH2A1.2 did not show any relationship with lung cancer progression. Therefore, it is presumed that macroH2A1.1 expression is limited to tumors showing a low proliferation index [38].

Sporn et al. also performed cell culture experiments that allowed for changes in macroH2A1 expression to be observed during cell differentiation. The researchers used Caco-2 cells as a cell model for CRC, which differentiate and polarize when cultured beyond confluency and in standard conditions [92]. They observed an up-regulation in macroH2A1.1 transcript and protein levels that reflected the degree of cellular differentiation. Conversely, macroH2A1.2 transcript levels were decreased, while the protein levels remained constant. Using pathway-focused qPCR analyses using PCR arrays, the researchers assessed the changes accompanying the increase in macroH2A1.1 levels during cellular differentiation. In differentiated cells, they found a global down-regulation of cell cycle markers crucial to all phases of cell cycle progression, together with a down-regulation of genes associated with checkpoint and DNA damage control, reflecting a state of cellular differentiation without proliferation. Only a few genes were up-regulated, including *CDKN1A, CDKN2B,* and *RBL2,* which are involved in cell cycle arrest, and *CCNG1* and *CCNG2,* which both have anti-proliferative activity [90]. MacroH2A1.1 knockdown in FET cells, a cell model obtained from an early stage human colon cancer, led to a phenotype of enhanced cell proliferation and DNA replication. Here, the cells showed increased expression of *HRC5, BRCA2, CCND2, HUS1, NBN*, and *CITED2*, which are genes involved in DNA replication, and *SERPINB2*, which is an apoptotic inhibitor. Knockdown of macroH2A1.2 led to a similar phenotype, explained by concomitant decrease in macroH2A1.1 levels following macroH2A1.2 knockdown [90]. Similar results were obtained in a murine model of lung cancer in which tumor senescence was induced by K-*Ras*G12V [38]. Here, IHC analyses showed an increase in macroH2A1.1 levels, corroborating its role as a hallmark for cellular senescence, and as a key component of senescence-associated heterochromatin foci (SAHF) [38]. Taken together these results are consistent with the idea that macroH2A1.1 marks cellular differentiation and cell senescence. Moreover, the two macroH2A1 splice variants have distinct functions in CRC progression (Figure 2).

### 5.3. MacroH2A2 in Anal SCC

The anal tract comprises the last part of the digestive system. The incidence of squamous cell carcinoma (SCC) of the anus has increased over the last three decades, most markedly in high-income countries, with a standard incidence of 4–8 per 100,000 people [93]. SCC usually originates from anal intraepithelial neoplasia (AIN), which is organized into three classes on the basis of its progression [94]. AIN usually occurs when clusters of abnormal cells accumulate from lesions in the mucosa of the anal canal; alternatively, it can also be caused by certain strains of the sexually transmitted human papillomavirus (HPV) or human immunodeficiency virus (HIV) [95,96].

MacroH2A2 histone has been implicated in AIN and SSC progression [97]. Hu et al. assayed macroH2A2 expression by IHC on malignant tissues derived from both AIN and SSC. They found that macroH2A2 expression was decreased in 38% of the AIN stage III samples and in 71% of the anal SSC tissues. Moreover, when investigating the recurrence of lesions in patients with AIN, Hu et al. found a significantly shorter time to recurrence in macroH2A2-negative patients than macroH2A2-positive patients. Moreover, the samples that showed a decreased level of macroH2A2 expression during IHC analysis also displayed a strong presence of HPV and HIV sequences in the genomic DNA, thus correlating macroH2A2 levels with viral infection. In conclusion, macroH2A2 expression is a predictor of a better survival rate in patients with AIN and SSC, thus, its expression may be assayed as a prognostic marker of anal neoplasm progression [97] (Figure 2).

## 6. Perspectives

This review has summarized the current knowledge regarding the roles of the histone variants macroH2A1 (with its 2 isoforms macroH2A1.1 and macroH2A1.2) and macroH2A2 as tumor suppressors or oncogenes in the GI tract. MacroH2A histones act in a context-dependent manner in different parts of the GI tract, but it is unclear whether DNA binding activity can be manipulated to revert tumorigenesis. In general, there is a lack of knowledge regarding the differential genomic distribution of endogenous macroH2A1.1/macroH2A1.2 and macroH2A2, due to the absence of suitable specific Chromatin Immunoprecipitation (ChIP)-grade antibodies. A comprehensive picture of the expression patterns of macroH2A histones in different GI cell types, normal or cancerous, is missing. Advancements in oncogenomics and CSC biology are now required to develop clinical and therapeutic applications that revolve around these macro histone variants.
